# Microbial approaches for targeting antibiotic‐resistant bacteria

**DOI:** 10.1111/1751-7915.12783

**Published:** 2017-08-03

**Authors:** Wing Fei Wong, Marina Santiago

**Affiliations:** ^1^ OpenBiome 200 Inner Belt Rd Somerville MA 02143 USA; ^2^ Finch Therapeutics 200 Inner Belt Rd Somerville MA 02143 USA

## Abstract

Antibiotic resistant bacterial infections are a global public health challenge that has been increasing in severity and scope for the last few decades. Without creative solutions to this problem, treatment of injuries and infections will become progressively more challenging. A better understanding of the human microbiome has led to a new appreciation for the role commensal microbes play in protecting us from pathogens, especially in the gut. Antibiotics lead to disruption of the gut microbial ecosystem, enabling colonization by antibiotic resistant bacterial pathogens. Many different lines of research have identified specific bacterial taxa and mechanisms that play a role in colonization resistance, and these lines of research may one day lead to microbial therapeutics targeting antibiotic resistant bacteria. Here, we discuss a few of these strategies and the challenges they will need to overcome in order to become an effective therapeutic.

## Antibiotic‐resistant bacteria pose a threat to public health

A major threat to Sustainable Development Goal 3 (ensuring healthy lives and promoting well‐being for all at all ages) is the rise of antibiotic‐resistant bacteria (ARB). Without new therapeutic strategies, the World Health Organization has stated that we could be headed for a ‘post‐antibiotic era’, in which previously treatable infections are once again deadly (WHO Media Cent, 2014). In the USA alone, the CDC estimates that more than 2 million people contract antibiotic‐resistant infections every year, resulting in more than 23 000 deaths (US Department of Health and Human Services, Centers for Disease Control and Prevention, 2013). This results in more than $20 billion in healthcare costs per year, with an additional $35 billion in lost economic output (Roberts *et al*., 2009; US Department of Health and Human Services, Centers for Disease Control and Prevention, 2013). On an individual patient level, this translates to increased costs, longer hospital stays and a higher likelihood of adverse events and death (Roberts *et al*., 2009; US Department of Health and Human Services, Centers for Disease Control and Prevention, 2013; Gandra *et al*., 2014).

## Microbial strategies for decreasing antibiotic resistance are promising and sustainable

The human microbiome, which consists of all the bacterial, fungal and viral microorganisms that colonize epithelial surfaces of the human body, may hold the key to fighting ARB. Members of the human microbiome play roles in many aspects of human development (Vaishnava *et al*., 2008; Dinan and Cryan, 2012; Sommer and Bäckhed, 2013; Peterson and Artis, 2014; De Santis *et al*., 2015; Chung *et al*., 2016). Generally, these organisms are commensal, but under certain conditions, some of these bacteria have been associated with chronic and acute disorders (Becattini *et al*., 2016; Nagao‐Kitamoto *et al*., 2016; Fung *et al*., 2017; Sommer *et al*., 2017; Wen and Duffy, 2017).

Antibiotic‐resistant bacterial infections are often a direct result of a disruption of the gut microbiome (Carlet, 2012; Sassone‐Corsi and Raffatellu, 2015; Pamer, 2016). A healthy intestinal microbiome consists of a highly diverse population. When this diversity is decreased by antibiotic treatment, niches become available for pathogen colonization. Domination of the gut microbiome by a pathogen places patients at high risk for developing infection by that pathogen as the gut barrier integrity weakens, enabling pathogen translocation across the epithelial barrier (Taur *et al*., 2012). Furthermore, ARB‐colonized patients can serve as vectors of ARB transmission.

**Figure 1:**
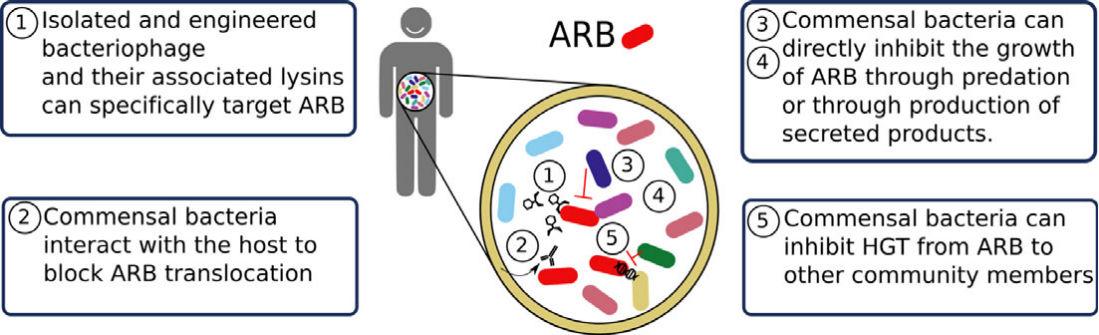
Microbial therapeutics target antibiotic‐resistant bacteria through many different mechanisms.

There are two major microbial strategies that are being pursued for combatting ARB. These include the following: (i) bacteriophage‐based strategies can target specific strains that colonize patients and cause chronic infections, and (ii) microbial remediation of the gastrointestinal tract relies on commensal bacteria for inhibition of ARB growth and transmission. In this review, we will discuss some exciting examples for each of these promising lines of research (Fig. 1).

## Bacteriophage precisely target ARB pathogens

Bacteriophage‐based therapies focus on using phage or its component proteins to target highly specific strains of bacteria. The approach of using bacteriophage (phage) isolates to treat bacterial infections has traditionally been pursued in the former Soviet Union and Eastern Europe (Sulakvelidze *et al*., 2001). These reports speak to the safety profile of this therapy (Weber‐Dabrowska *et al*., 2000; Bruttin and Brüssow, 2005). In addition, phage are readily modifiable to combat emergence of newly arising bacterial threats (Samson *et al*., 2013). However, current research into whole phage therapies lacks rigorous proof of efficacy, namely properly conducted randomized placebo‐controlled studies. There are concerns over immunogenicity of phage therapy, as well as development of bacterial resistance to bacteriophages (Lu and Koeris, 2011; Pires *et al*., 2016). Furthermore, requirement of regulatory approval in the United States, among other obstacles, prevents widespread use of phage therapy.

Issues of using whole phage therapy may be mitigated by using bacteriophage lysins: phage‐encoded peptidoglycan hydrolases that induce rapid lytic death (Young, 1992; Young and Bläsi, 1995; Wang *et al*., 2000; Borysowski *et al*., 2006). Exogenous recombinant lysins effectively target Gram‐positive bacteria, as there is no outer membrane to prevent access to the cell wall (Loeffler *et al*., 2001; Fenton *et al*., 2010). Lysins are also reported to have narrow host range, which theoretically spares the surrounding commensal microflora (Fenton *et al*., 2010). However, lysins face similar therapeutic challenges as phage therapy: like all other foreign agents, the host will develop neutralizing antibodies, which will reduce the levels of enzyme during treatment. Furthermore, this therapeutic is largely ineffective in Gram‐negative bacteria. While this may be circumvented using outer membrane permeabilizers, there may be cytotoxic effects associated with this approach that limit its safety (Amaral *et al*., 2007; Walmagh *et al*., 2013).

Advances in genetic engineering technology have allowed researchers to manipulate phage to enhance antibacterial activity, targeting and delivery. Engineered phages can deliver genes conferring increased sensitivity to antibiotics (Lu and Collins, 2009; Edgar *et al*., 2012), disrupt biofilm matrices through delivery of biofilm‐degrading enzymes (Lu and Collins, 2007) and deliver lethal‐agent phagemid particles (Westwater *et al*., 2003). Using the CRISPR‐Cas system, RNA‐guided nucleases are delivered via phagemids into bacterial cells, where they target specific genetic sequences and induce a double‐strand break, leading to plasmid loss or cell death (Citorik *et al*., 2014). While engineered phage therapy is promising, more research is required for optimizing vector delivery and minimizing immunogenicity.

## Microbiome restoration inhibits ARB growth and transmission

Commensal bacteria can provide resistance to ARB by interacting with the host. For example, some Gram‐negative obligate anaerobes are known to induce the production of host antimicrobial peptides (Sonnenburg *et al*., 2006; Brandl *et al*., 2008; Kinnebrew *et al*., 2010; Ubeda *et al*., 2013). In addition, short‐chain fatty acid (SCFA) production is intimately involved in pathogen defence. SCFAs are the main source of energy for colonocytes, induce IgA production, reduce inflammation and may be involved in increasing the thickness of the mucus layer (Zimmerman *et al*., 2012; Earle *et al*., 2015; Desai *et al*., 2016; Jones, 2016; Wu *et al*., 2016; Goverse *et al*., 2017; Olsan *et al*., 2017; Rowland *et al*., 2017). These strains are vital for preventing bacterial translocation by reinforcing the gut barrier.

Other commensal bacteria can directly attack or inhibit pathogen growth. In fact, co‐culture of some commensal and pathogenic strains results in the direct killing of the pathogen through the production of secreted molecules like bacteriocins (Gilmore *et al*., 2015; Gaca and Gilmore, 2016; Sassone‐Corsi *et al*., 2016). Strains producing these molecules could be used therapeutically to eliminate populations of ARB from the gut.

Predatory bacteria have the potential to play a role in the management of antibiotic‐resistant infections. *Bdellovibrio* spp. and *Micavibrio* spp. are proteobacteria whose life cycle contains an attack phase where they attach to, or invade and kill other bacteria. These predators can kill many Gram‐negative pathogens, including those resistant to antibiotics of last resort (Markelova, 2010; Kadouri *et al*., 2013)*. In vivo* experiments with predatory bacteria have established their safety and efficacy at decreasing pathogen burden in mammalian models (Westergaard and Kramer, 1977; Shatzkes *et al*., 2015, 2016; Boileau *et al*., 2016; Zurawski *et al*., 2017). However, one concern is that the observed efficacy is not a result of direct pathogen inhibition, but rather indirect activation of the immune system in surrounding tissues. Future studies assessing safety to the human host and microbiome will shed light on the utility of this approach.

Commensal organisms may be able to combat antibiotic resistance through inhibition of horizontal gene transfer (HGT). Antibiotic resistance genes are often found on mobile genetic elements like plasmids and transposons, which travel to other bacteria through HGT. Commensal microbes may be able to prevent this process; a consortium of four anaerobic bacterial strains can suppress mobilization of KPC, a common beta‐lactamase gene, from *E. cloacae* to *K. pneumoniae* in a germ‐free mouse model (Nudel *et al*., 2017).

These mechanisms, among others, may contribute to the success seen using faecal microbiota transplants (FMTs) for decolonization of ARB. FMT has been used extensively for treatment of recurrent *Clostridium difficile* infections with a remarkably high efficacy rate (85%; Drekonja *et al*., 2015). FMT is thought to act by delivering commensals that (i) directly compete for niches with *C. difficile*, (ii) convert primary bile acids, which are required for *C. difficile* spore germination, into secondary bile acids, and (iii) activate the immune system and help maintain the gut barrier, reducing bacterial translocation across the epithelial layer and preventing pseudomembranous colitis (Khoruts and Sadowsky, 2016).

Faecal microbiota transplant success in the context of *C. difficile* has led to the reporting of many small case studies assessing the efficacy of FMT for decolonization of other ARB. To date, 18 studies with a total of 101 patients have been published that use FMT to decolonize some of the most concerning ARB (Freedman, 2014; Jang *et al*., 2014; Singh *et al*., 2014; Lagier *et al*., 2015; Lombardo, 2015; Nancy *et al*., 2015; Stripling *et al*., 2015; Wei *et al*., 2015; Biliński *et al*., 2016; Dubberke *et al*., 2016; García‐Fernández *et al*., 2016; Jouhten *et al*., 2016; Millan *et al*., 2016; Smith, 2016; Sohn *et al*., 2016; Bilinski *et al*., 2017; Davido *et al*., 2017; Ponte *et al*., 2017). A pooled analysis of these data shows that 82% of patients were found to be decolonized or have a significantly reduced ARB load after FMT (Table 1). Unfortunately, while patients with *C. difficile* infection are willing to accept FMT treatment, patients who are simply colonized with ARB are asymptomatic and therefore may be less willing to be treated with FMT. Furthermore, scaling up of FMT manufacture to treat the millions of people around the world colonized with ARB would be incredibly challenging. In the future, we expect that microbial therapeutics for ARB will more closely resemble over‐the‐counter probiotics, but to bring these to the clinic, we will need more detailed mechanistic information on how FMT exerts its effect against ARB. Fortunately, recent work has shed some light on specific consortia that are able to decolonize ARB (Caballero *et al*., 2017).

**Table 1:** FMT decolonizes antibiotic‐resistant bacteria from the human gut

Report	# Patients	VRE	CRE	ESBL‐E	Others	Results
Freedman, 2014	1		X			1/1 decolonized for at least 8 months
Singh, 2014	1			X		1/1 decolonized at 2 weeks
Stripling, 2015	1	X				1/1 reduced relative abundance and no further VRE infections for 1 year
Crum‐Cianflone, 2015	1	X	X		X	1/1 reduced MDRO colonization and no episodes of sepsis for 2 years
Jang, 2014	1	X				0/1 decolonized at 3 months
Lombardo, 2015 (SER‐109)	8	X				8/8 titers decreased > 2 fold at 4 weeks
Bilinski, 2016	1		X	X		1/1 decolonized at 10 days
Lagier, 2015	1		X			1/1 decolonized at 7 days
Wei, 2015	5				X	5/5 decolonized of MRSA for 3 months
Eysenbach, 2016	9	X				9/9 decolonized at first time point measured post‐FMT
Dubberke, 2016	11	X				8/11 decolonized at last available follow‐up
Jouhten, 2016	8	X	X	X	X	8/8 reduction in number and diversity of antibiotic resistance genes
Millan, 2016	20		X	X	X	20/20 reduction in number and diversity of antibiotic resistance genes
Garcia‐Fernandez, 2016	1		X	X		1/1 decolonized at 6 weeks
Sohn, 2016	3	X				0/3 decolonized for 3 months
Davido, 2017	8	X	X			2/6 CRE decolonized at 1 month, 1/2 VRE decolonized at 3 months
Ponte, 2017	1		X			1/1 CRE decolonized at 15, 45, and 100 days
Bilinski, 2017	20	X	X	X	X	15/20 decolonized at 1 month
Total:	101	38/46 (83%)	45/57 (79%)	50/54 (93%)	39/39 (100%)	83/101 (82%) decolonized or decreased in antibiotic resistance genes at primary endpoint

VRE, Vancomycin Resistant Enterococeus; CRE, Carbapenem Resistant Enterobacteriaceae; ESBL‐E, Extended Spectrum Beta Lactamase Producing Enterobacteriaceae; FMT, fecal microbiota transplant.

Unless otherwise noted, patients were treated with fecal microbiota transplant. Some patients were co‐colonized with multiple pathogens.

## Future outlook: Microbial technology will likely support traditional therapeutic approaches

Microbiome‐based strategies for decreasing antibiotic resistance are appealing for three reasons. First, unlike antibiotic treatment, they do not disrupt the microbial ecosystem, decreasing the risk of side‐effects and development of chronic issues associated with a disrupted microbiome. Second, microbial approaches do not select for additional antibiotic resistance genes, increasing the life of available antibiotics. Finally, ARB decolonization decreases transmission rates, preventing infection in both the decolonized patient and the surrounding community.

However, there are a few obstacles to bringing microbial approaches to the clinic. Although methods for fermentation of large bacterial cultures for manufacturing probiotics exist, culturing anaerobic bacteria at large scales is likely to be significantly more challenging. Second, the microbiome field is still quite young, and there is still uncertainty about which mechanisms of ARB elimination and which bacterial strains themselves will be the safest and most effective. Therefore, the first microbial therapeutics targeting ARB will likely be complex communities derived from whole stool, such as RBX2660 from Rebiotix, SER109 from Seres and FIN‐403 from Finch Therapeutics.

For now, it seems unlikely that microbial approaches will be able to outcompete traditional small‐molecule antibiotic approaches for treating infections. Small‐molecule antibiotics are very inexpensive and, although resistance is rising, are still remarkably efficacious. However, as resistance becomes more prevalent, novel strategies will be needed to increase the life of available antibiotics. Along with other creative solutions, microbial approaches that can treat infections and/or eradicate ARB pathogens from the gut are likely to be valuable additions to our anti‐infective arsenal. While there are still many questions that need to be answered before microbial products targeting antibiotic resistance reach the clinic, we are excited for the future of this very promising field of technology.

## Conflict of Interest

None declared.
